# Development and verification of a 10K liquid chip for Hainan black goat based on genotyping by pinpoint sequencing of liquid captured targets

**DOI:** 10.1186/s12863-024-01228-8

**Published:** 2024-05-07

**Authors:** Yong Meng, Wencan Zhang, Yiwen Cheng, Yanru Wu, Haotian Wu, Meirong He, Si Chen, Churiga Man, Hongyan Gao, Li Du, Qiaoling Chen, Fengyang Wang

**Affiliations:** Hainan Key Laboratory of Tropical Animal Reproduction & Breeding and Epidemic Disease Research, Engineering Key Laboratory of Haikou, School of Tropical Agriculture and Forestry, Hainan University, Haikou, 570228 Hainan Province China

**Keywords:** Hainan black goat, Targeted sequencing, Genotyping, Liquid chip

## Abstract

**Background:**

China has thousands years of goat breeding and abundant goat genetic resources. Additionally, the Hainan black goat is one of the high-quality local goat breeds in China. In order to conserve the germplasm resources of the Hainan black goat, facilitate its genetic improvement and further protect the genetic diversity of goats, it is urgent to develop a single nucleotide polymorphism (SNP) chip for Hainan black goat.

**Results:**

In this study, we aimed to design a 10K liquid chip for Hainan black goat based on genotyping by pinpoint sequencing of liquid captured targets (cGPS). A total of 45,588 candidate SNP sites were obtained, 10,677 of which representative SNP sites were selected to design probes, which finally covered 9,993 intervals and formed a 10K cGPS liquid chip for Hainan black goat. To verify the 10K cGPS liquid chip, some southern Chinese goat breeds and a sheep breed with similar phenotype to the Hainan black goat were selected. A total of 104 samples were used to verify the clustering ability of the 10K cGPS liquid chip for Hainan black goat. The results showed that the detection rate of sites was 97.34% -99.93%. 84.5% of SNP sites were polymorphic. The heterozygosity rate was 3.08%-36.80%. The depth of more than 99.4% sites was above 10X. The repetition rate was 99.66%-99.82%. The average consistency between cGPS liquid chip results and resequencing results was 85.58%. In addition, the phylogenetic tree clustering analysis verified that the SNP sites on the chip had better clustering ability.

**Conclusion:**

These results indicate that we have successfully realized the development and verification of the 10K cGPS liquid chip for Hainan black goat, which provides a useful tool for the genome analysis of Hainan black goat. Moreover, the 10K cGPS liquid chip is conducive to the research and protection of Hainan black goat germplasm resources and lays a solid foundation for its subsequent breeding work.

**Supplementary Information:**

The online version contains supplementary material available at 10.1186/s12863-024-01228-8.

## Background

Domestic goats (*Capra hircus*) are distributed in five continents and have successfully adapted to various climates, such as deserts, mountains and tropical regions. China has a long history of goat production and abundant genetic resources. At present, there are about 138 million goats of 58 local breeds in China [1]. The various genetic resources are an important part of the global biological genetic resources diversity and provides good materials not only for improving local breeds but developing new breeds [2]. In recent years, due to social development and environmental change, the gene pool of Chinese native goat breeds is in danger [3]. The Hainan black goat is a high-quality local goat breed in Hainan Province, China. It has delicious meat and resistance to high temperature, humidity and disease. However, its reproductive performance and milk yield are poor [4]. Accordingly, it is particularly important to excavate the genes that relate with their excellent traits. For example, the growth differentiation factor 9 (GDF9) had a C / T missense mutation at 2541 bp in the 3 ' segment, which was significantly correlated with the number of first-born lambs [5, 6]. It is helpful to improve the reproductive efficiency and growth performance of Hainan black goat and increase its economic benefits. Therefore, it is an important task at this stage to protect the genetic resources of Hainan local black goat, improve its breeds and cultivate new breeds. To realize that, molecular marker-assisted breeding technology is an effective way.

Genomic selection (GS) is an important method for genetic improvement of economic traits in livestock and poultry [7]. Genetic variation detection technology based on molecular markers is a very good molecular detection method [8]. DNA molecular marker technology has been widely used in DNA fingerprinting, genetic diversity, population structure analysis and marker-assisted breeding [9]. With the development of high-throughput sequencing and array technology, the cost of large-scale genotyping has been greatly reduced. In addition, the selection of SNP as genetic marker has become a trend. At present, there are three main methods for large-scale SNP genotyping [10]: genotyping by sequencing (GBS), whole genome (re) sequencing (WGS) and array-based methods. SNP chips, also known as SNP arrays, are used for SNP genotyping. They are widely used in diversity analysis, quantitative trait sites (QTL) mapping, tracking introgression, genetic resource development and DNA fingerprinting [11, 12]. Genome-wide SNP chips with different densities are widely used in animals. For goats, Tosser-Klopp et al. launched a goat medium density 52 K BeadChip [13] called GoatSNP50 [14], which is the most commonly used goat SNP chip. In 2017, Qiao et al. successfully designed a medium-density genome-wide target enrichment-aided chip for cashmere goat [15]. However, SNP chips designed for large commercial breeds are not the best choice for diversity research and genetic evaluation of local breeds. They also cannot be used to maintain breed-specific genetic characteristics [16]. At present, there is no SNP chip available for Chinese tropical goats. It is necessary to customize a SNP chip for Hainan black goat to evaluate its germplasm resources and perform DNA fingerprinting. Thus, we can better excavate and protect the valuable genetic resources of Hainan black goat.

**Fig. 1:**
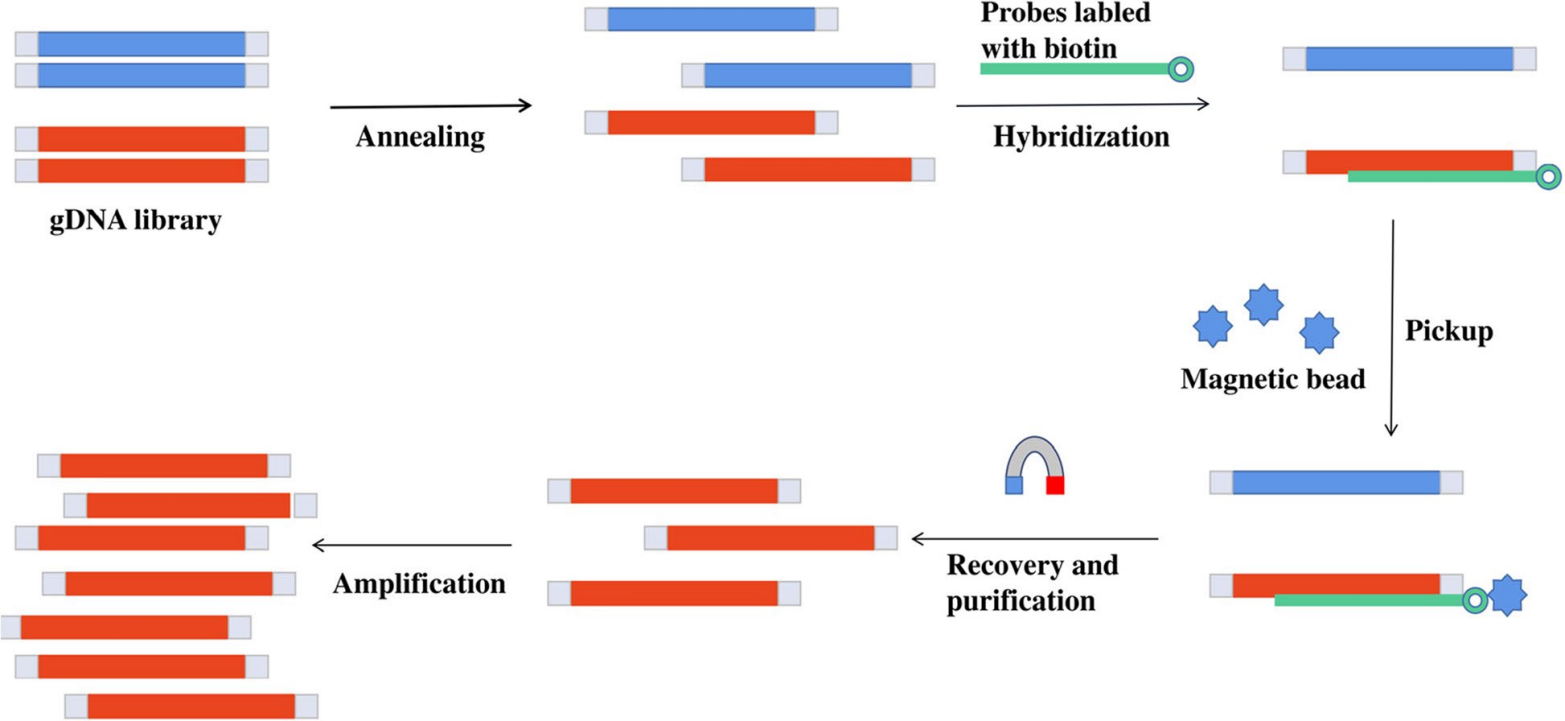
Principles and processes of cGPS technology

In order to simplify genome sequence analysis, specific nucleic acids are captured, targeted or enriched by other ways so as to analyze the sequence of interest in the genome to a greater depth [17]. Recently, there has been significant progress in target sequencing and in-solution capture [18]. For example, liquid chips for animals include sheep [19] and chicken [20]. Genotyping by pinpoint sequencing of liquid captured targets is a targeted sequencing genotyping technology. It is also a targeted sequencing based on solution hybridization. The main process diagram of cGPS technology is shown as follows (Fig. 1). We designed the liquid chip of Hainan black goat based on cGPS to meet the needs of SNP or insertion and deletion (InDel) genotyping in the genome region of interest.

At present, there is an inevitable trend to carry out the research of goat genomics in China. We developed a 10K liquid chip for Hainan black goat, which can be used for breeding and provenance identification. The chip is suitable for the evaluation and analysis of germplasm resources of different goat breeds. It can be used to efficiently and quickly analyze the pedigree of Hainan black goat population at a low cost. Moreover, the chip can promote the protection of Hainan black goat germplasm resources, provide an important tool for the subsequent marker-assisted breeding of Hainan black goat and the study on the molecular mechanism of important traits.

## Result

### Results of whole-genome resequencing data analysis in goats

A total of 87 goat genomic data was obtained from the resequencing of Hainan black goats and public databases of other goat breeds. After filtering, the clean reads were aligned with the reference genome, then the average alignment rate, coverage rate and sequencing depth of each sample were analyzed. The results showed that the resequencing data was of good quality and could be used for further analysis. In addition, 88,454,696 SNPs were identified in 87 goats. (Additional file 1: Table S1).

### Candidate SNP sites from whole-genome resequencing

**Fig. 2:**
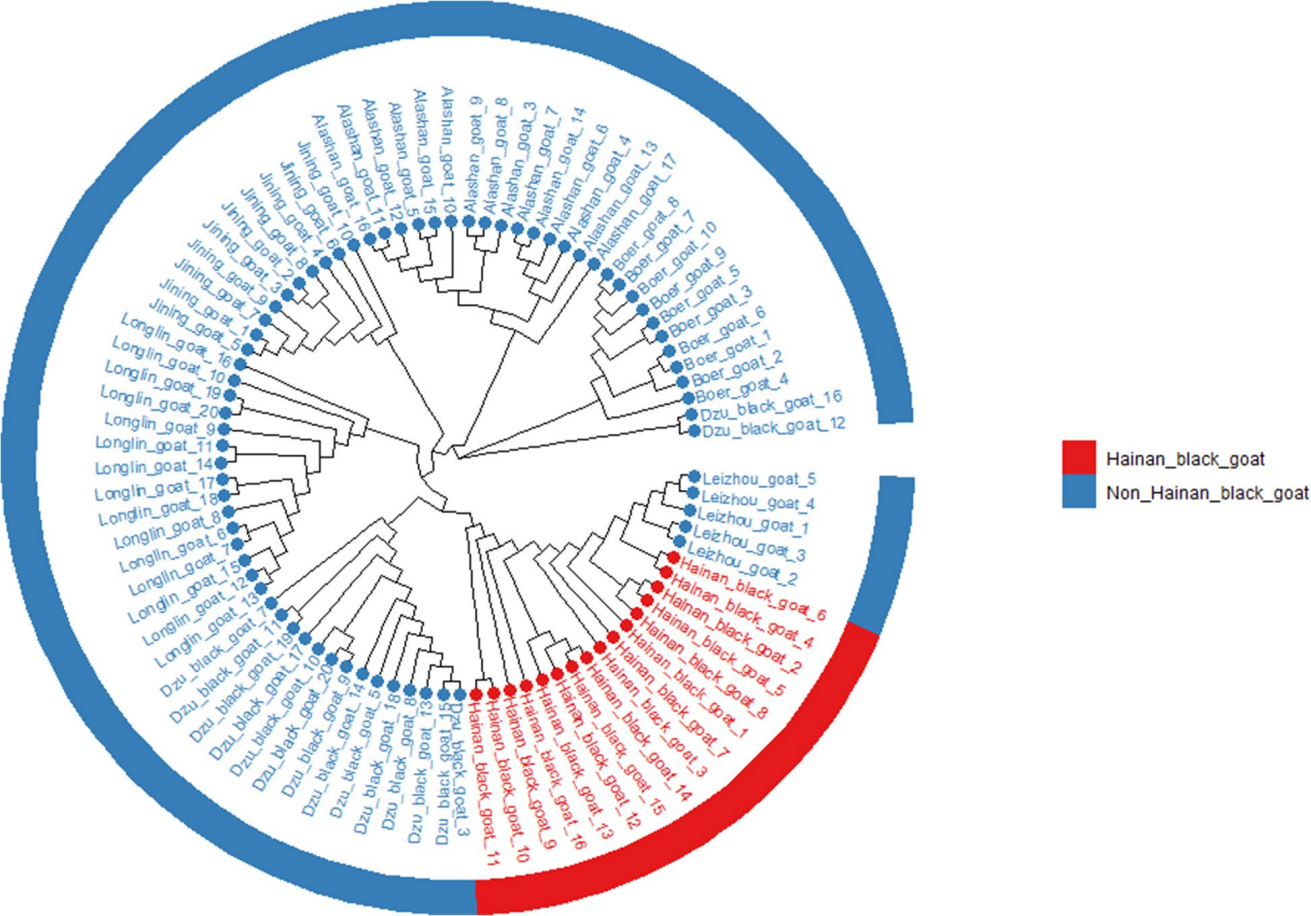
Phylogenetic tree of 87 resequenced goat samples. Red represents the Hainan black goat population and blue represents the non-Hainan black goat population

In order to obtain the SNP loci available in the re-sequencing data, 1212378 SNPs were obtained after filtering for the construction of the development tree. The results (Fig. 2) showed that the reference population of the Hainan black goat and the non-Hainan black goat were successfully constructed, which included 16 Hainan black goats and 71 other goats (included 15 Longlin goats, 5 Leizhou goats, 16 Dazu black goats, 15 Alxa cashmere goats, 10 Jining grey goats and 10 Boer goats), respectively. The phylogenetic tree also showed that Hainan black goats were closely related to Leizhou goats. Next, the Fst values and polymorphisms of all SNP sites in both reference populations were determined. Thirty-nine thousand one hundred one candidate SNP sites with high polymorphism in both Hainan black goat and non-Hainan black goat populations were screened from the resequencing data (Additional file 2: Table S2). There were 1,530 candidate SNP sites with Fst > 0.5 in both reference populations (Additional file 3: Table S3).

### Candidate SNP sites from GGVD database

A total of 2,514 high polymorphic (MAF > 0.05) SNP sites (Additional file 4: Table S4) and 125 immunogene SNP sites (Additional file 5: Table S5) were derived from GGVD. The immunogenes included *IL6*, *TNF*, *IL1B*, *IL10*, *IFNG* and other 34 genes of interest.

### SNP candidate sites from literature sources

Literatures on SNPs that associated with important traits, including meat quality, reproduction, growth, production, disease resistance, and immunity in goats and sheep, were searched and browsed in PubMed and CNKI (China National Knowledge Infrastructure). SNP sites information in more than 270 Chinese and foreign literatures was recorded. Then, the flanking sequences of the SNP sites were searched and aligned with ARS1 to relocate their position on ARS1. After testing, a total of 2,035 candidate SNP sites related to important traits were eventually determined (Additional file 6: Table S6).

### Design results of 10K cGPS liquid chip for Hainan black goat

**Fig. 3:**
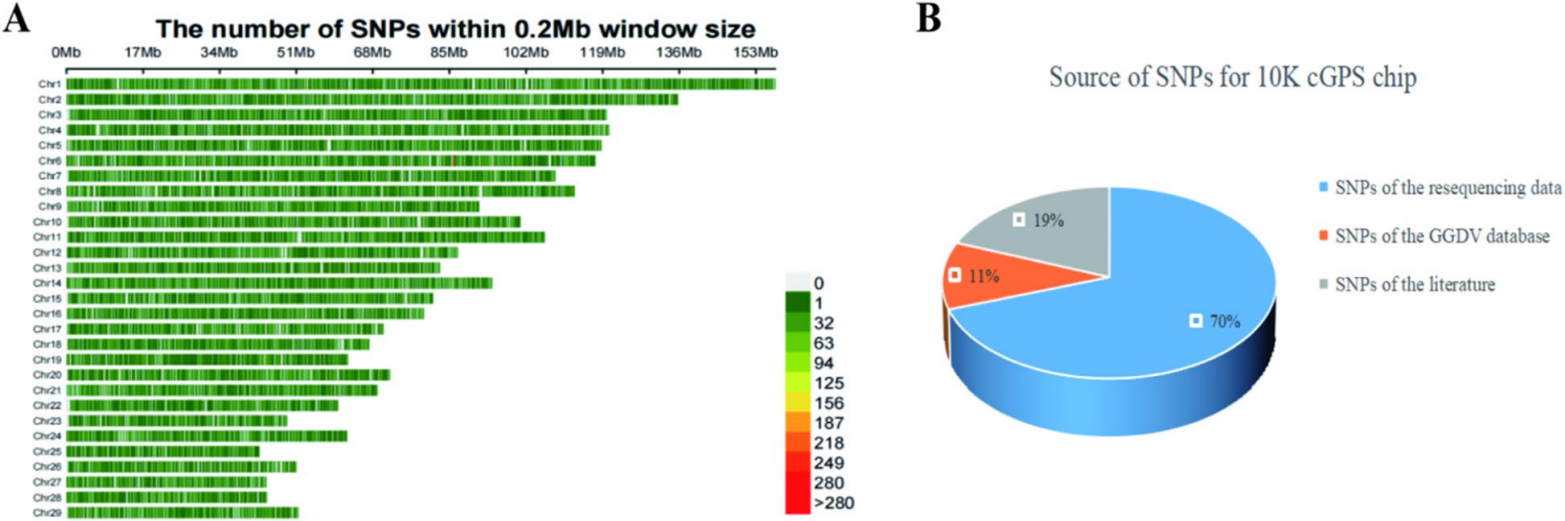
Characterization of SNP sites on the 10K cGPS liquid chip for Hainan black goat. **A** The number of SNPs within 0.2 Mb window size. **B** Source of SNPs for 10K cGPS chip

In order to use the data for probe design and synthesis, the repetitive sites were removed, and the sites and sequences were all converted into information consistent with the goat reference genome (ARS1) (chromosome, physical location, sequence, reference genome genotype). Then, this information was used for the design and synthesis of the probe. Among the 45,588 candidate SNP sites, 10,677 qualified sites were screened according to the screening requirements and probe design results (Additional file 7: Table S7). The distribution map (Fig. 3A) of 10,677 sites in the reference genome showed that the sites on the chip were basically evenly distributed in autosomes. The sources of SNP sites on the 10K cGPS liquid chip were showed in picture (Fig. 3B). Among them, the sites from resequencing data accounted for approximately 70%, including 6,629 high polymorphic sites and 803 sites with Fst value greater than 0.5 in the reference population of Hainan black goat and non-Hainan black goat. The percent of sites form GGVD was about 11%, which contained 1,136 high polymorphic sites and 75 sites in immunogenes of interest. Besides, there were 2,034 sites related to important traits from the literature, accounting for about 19%. Eventually, 7,765 high polymorphic SNP sites were selected in the final panel, which was about 72.7% of the total panel sites. These high polymorphic sites can be applied to the genotype analysis of different populations. Among the total screened sites, 9,100 (about 85.2%) were found in GGVD.

**Fig. 4:**
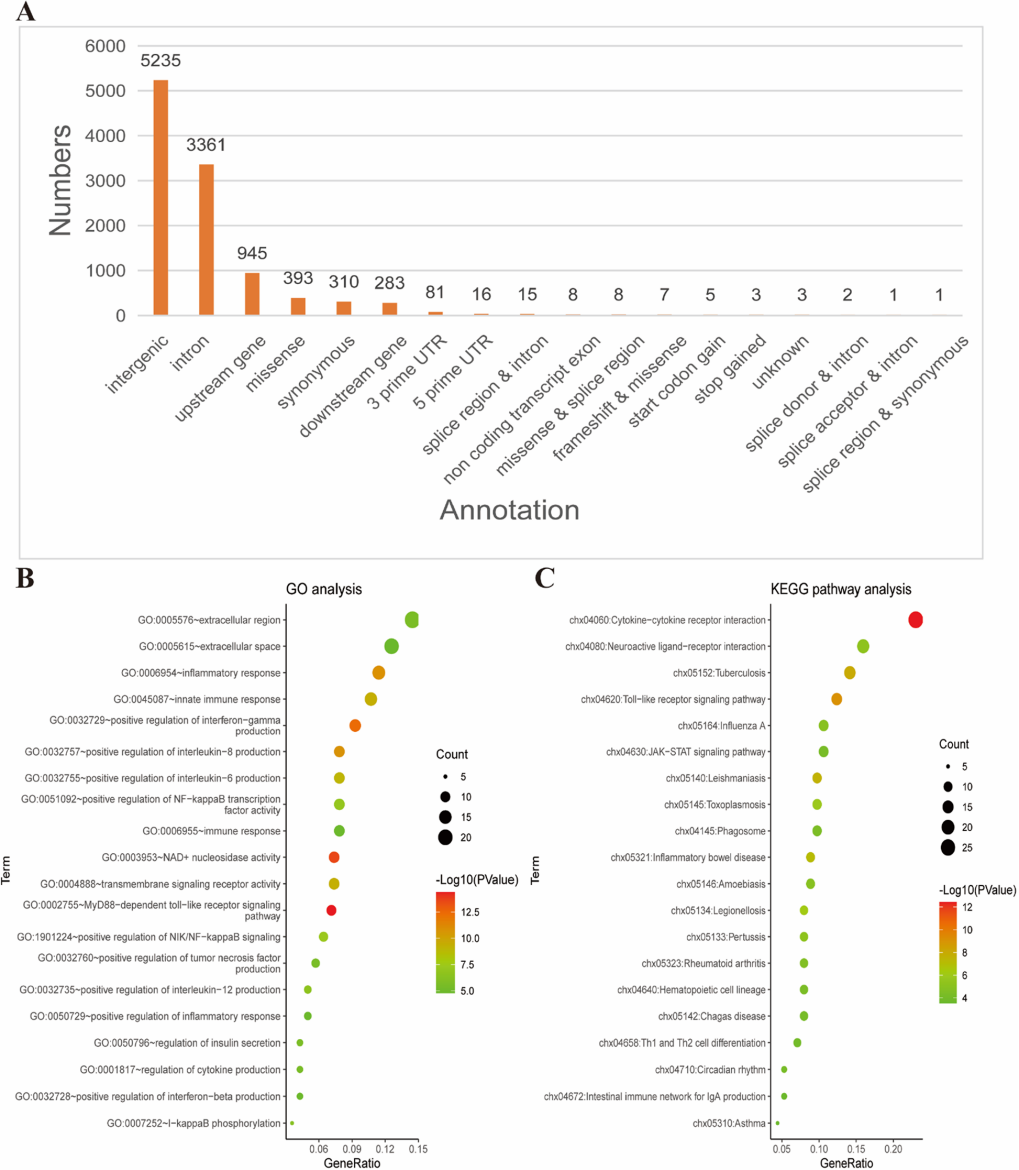
Evaluation of SNP sites on the 10K cGPS liquid chip for Hainan black goat. **A** Annotation of SNP sites on the 10K cGPS liquid chip for Hainan black goat. **B** Functional enrichment analysis and functional annotation of genes

Due to the short distance between some of the 10,677 SNP sites, the sites with a distance of no more than 100 bp can share a probe. In order to form the system of 10K cGPS liquid chip for Hainan black goat, a total of 10,571 probes were designed, which can capture 9,993 intervals. The annotation results of chip site (Fig. 4A) showed that most SNPs (49.02%) were between genes, 31.48% were in intron regions, and only 19.5% were located in other regions (Additional file 8: Table S8). Further, genes annotated with SNPs (Fst > 0.5) affected by moderate or high mutations were selected. Using David database [21] to find the data of gene enriched pathways, the results showed that GO and KEGG enriched terms were mainly immune related (Fig. 4B and C). This liquid chip is beneficial for searching potential immune related SNPs in Hainan black goat.

### Verification of 10K cGPS liquid chip for Hainan black goat

In order to verify the site detection rate of Hainan black goat 10K cGPS chip. A total of 104 genomic DNA samples were tested. The call rate was 97.34% -99.93%, and 84.5% of the SNP sites were polymorphic. The heterozygosity rate was 3.08%-36.80%. It can be seen that the site detection rate of the chip was very high and met the requirements (Additional file 9: Table S9).

**Fig. 5:**
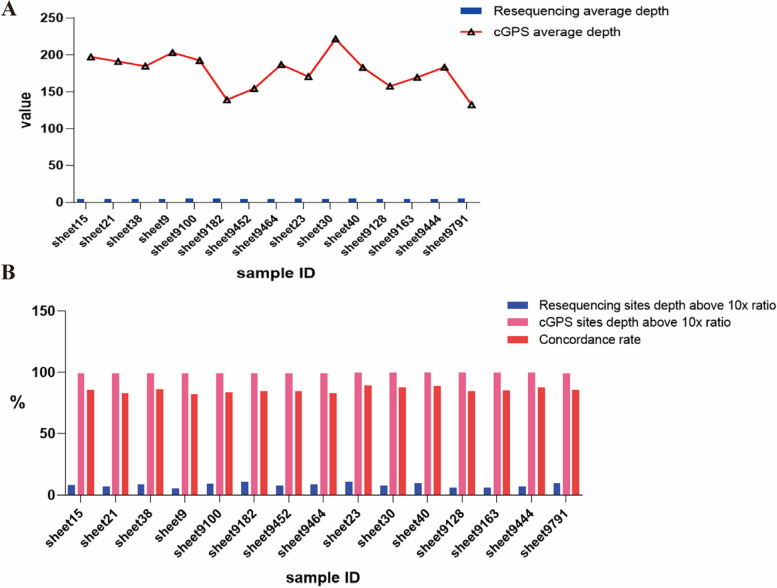
Average depth of sites, proportion of sites with depth above 10x, concordance rate between resequencing and cGPS detection results. **A** Average depth of sites between resequencing and cGPS detection results. **B** proportion of sites with depth above 10x, concordance rate between resequencing and cGPS detection results

In order to verify the consistency of the genotyping results of the chip, we used 15 resequenced DNA sample for genotype detection by the chip. Then, the genotyping results from cGPS liquid chip were compared with those from resequencing (Additional file 10: Table S10). The consistency rate was between 81.97% and 89.16%, and the average consistency rate was 85.58% (Fig. 5B). The average depth of samples in resequencing was low, which was 4.77. While the average depth of samples in cGPS liquid chip was 177.90 (Fig. 5A). The proportion of resequencing sites with depth of more than 10X was only 8.19%. In comparison, the proportion of cGPS sites with depth of more than 10X was 99.36% (Fig. 5B). Accordingly, there were some errors in the determination of genotypes by different sequencing depth.

In order to verify the repeatability of the 10K cGPS liquid chip for Hainan black goat, we selected four samples of GZHSY-10, sheet23, sheet30 and sheet9128 to compare the repeated detection results of the same genotype (Additional file 11: Table S11). The comparison of genotyping results in each sample showed that the consistency rate was between 99.66% and 99.82%. The average consistency rate was 99.75%, which showed the good repeatability of the chip (Table 1).

**Table 1:** Statistical table of genotype concordance rate in duplicate samples

Sample ID	Number of discordant SNPs	Concordance rate
GZHSY-10&GZHSY-10-re	19	99.82%
sheet-23&sheet-23-re	28	99.74%
sheet-30&sheet-30-re	22	99.79%
sheet-9128&sheet-9128-re	36	99.66%

**Fig. 6:**
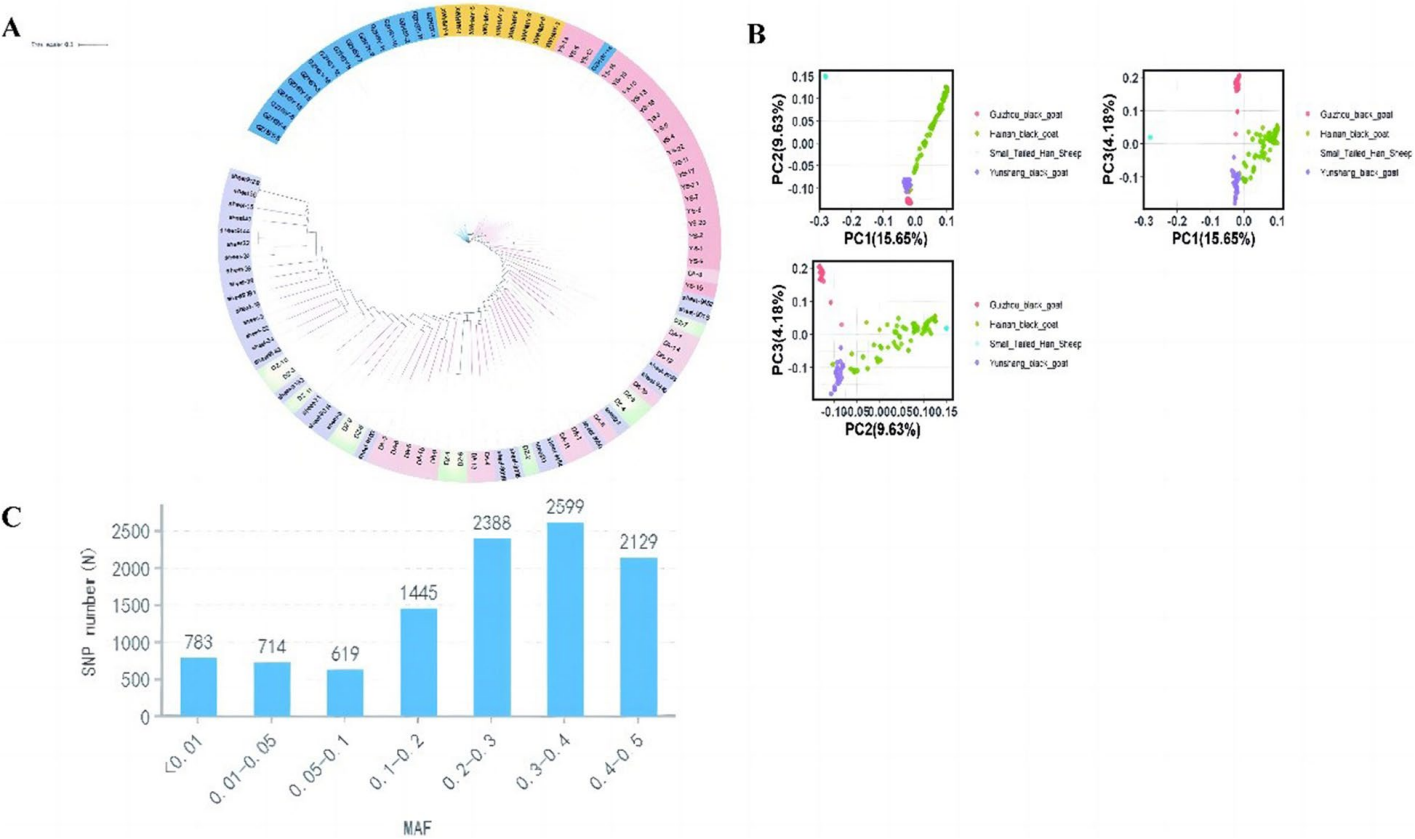
Phylogenetic tree, PCA analysis, and MAF statistical results of all SNP sites of the 10K cGPS liquid chip for Hainan black goat. **A** Phylogenetic tree of the chip detection results for 104 goat DNA samples. Pink, purple and green represent Hainan black goats in different regions of Hainan Province, respectively. Rose red represents Yunsahng (YS) black goat. Light blue represents Guizhou (GZ) black goat, and yellow represents Small-tailed Han (XWH) sheep. **B** PCA analysis of chip detection results for 104 goat DNA samples. Green dots represent Hainan black goats. Red dots represent Guizhou black goats. Blue dots represents Yunsahng black goat. And cyan dots represents Small-tailed Han sheep. **C** MAF statistics of the all SNP sites detected by the chip

In order to verify the clustering ability of the 10K cGPS liquid chip for Hainan black goat, cluster analysis was performed based on the test results of 104 samples. The results of phylogenetic tree and PCA showed that Hainan black goats had obvious clustering with other goat breeds and there were obvious clusterings among different goats, which basically realized the clustering function (Fig. 6A, B). Small-tailed Han sheep, a breed of sheep, can also be distinguished by the chip. We also found that the Hainan black goat in different regions of Hainan were not clustered but mixed with each other, which was related to High genetic diversity of Hainan black goats. It was worth noting that a Guizhou black goat and a Hainan black goat were mixed in the marginal clustering area of Yunshang black goat, which was related to the cultivation method of the new breed of Yunshang black goat. After 5 generations of research in 22 years, Yunshang black goat was cultivated by comparing the genes of different goat breeds around the world. The local Yunling black goat was used as the female parent and the Egyptian Nubian black goat was used as the male parent [22]. It is the first new breed of meat black goat in China developed by artificial breeding techniques. Therefore, we speculated that the genome of Yunshang black goat may contain the dominant genotypes of Guizhou black goat and Hainan black goat.

Finally, we summarized the detection results of all 104 samples and 4 repeated detection results, which reached up to 108. The genotyping results of SNP sites detected by the chip were obtained (Additional file 12: Table S12). Among the 108 chip detection results, the MAF, deletion rate, heterozygosity rate, and Fst value of all SNP sites were counted (Additional file 13: Table S13). The distribution map of MAF sites showed that the number of SNP sites was the most from 0.3 to 0.4, while the least between 0.05 and 0.1. The MAF of most SNP sites was higher than 0.01, and the proportion of SNP sites that met the requirements was 92.67%. However, there were 783 SNP sites with MAF value less than 0.01 (Fig. 6 C). Therefore, it was necessary to expand the sample size and adjust the SNP sites of the cGPS liquid chip.

## Discussion

Single nucleotide polymorphism (SNP) is widely used in genetic research and molecular breeding [23]. The selected SNP sites of the 10K cGPS liquid chip for Hainan black goat is divided into three parts. The first part is 40,631 SNPs from the whole genome resequencing of 7 representative goat breeds. Advances in whole genome sequencing technology help to discover SNPs [24]. We selected goat breeds from different regions of China, including Hainan black goats, as well as an abroad goat breed. The SNP sites from whole genome resequencing included 39,101 SNPs with high polymorphism in all goats and 1,530 SNPs with Fst > 0.5 in the populations of Hainan black goat and non-Hainan black goat. The 10K cGPS liquid chip is a customized SNP chip designed for Hainan black goat. The sites with high Fst value can be used to distinguish the genotypes of Hainan black goat and non-Hainan black goat. Huanhuan Fan et al. measured the Fst values and the heterozygosity of all SNP sites in the reference populations of sika deer and red deer. And 1,000 SNP sites with high Fst values were screened to form a 1 K sika deer SNP chip [25]. High polymorphism sites can be applied to analyze the genotype of different goat populations. When developing Eucalyptus EUChip60K chip, Orzenil B Silva-Junior et al. retained polymorphic SNPs between and within species, including those fixed in one specie but polymorphic relative to another species [26].

The second part is 2,639 SNPs from GGVD. Among the comprehensive databases that contain goat SNP information, dbSNP [27] and EVA [28] establish a compatible global system to assign unique identifiers for all submitted genetic variations and share the variation data of multiple species. However, dbSNP now only updates human variation information. In contrast, GGVD is more easier to use. The allele frequency data in GGVD will provide convenience for population genetic research and molecular marker design in goat breeding projects [29]. Besides, Animal-ImputeDB (http://gong_lab.hzau.edu.cn/Animal_ImputeDB/) is also a good choice. It provides not only genetic variation information of 13 animal species, but also online genotype interpolation, which will greatly promote animal genome selection and genetic improvement research [30]. Genetic variation of immunogene may play an important role in the susceptibility of a series of common diseases with inflammatory reaction [31]. Therefore, we selected the SNP sites of immunogenes. It has been reported that the SNP of *TNF-α* affects the reproductive performance and immune function of dairy cows [32]. *TLR2* plays an important role in the recognition of Gram-positive bacteria by innate immune system. The polymorphism of *TLR2* in goats may be related to the elevated somatic cell count in milk caused by mastitis [33]. Due to the strong disease resistance of Hainan black goat, we specifically searched for the SNP sites of some immunogenes in GGVD, which were of interest in our previous study. These sites were helpful to the subsequent mining of disease resistance genes of Hainan black goat.

The third part is 2,367 SNPs from the literature. Goats and sheep can be considered to have a common evolutionary origin [34]. We searched SNP sites that associated with important traits such as meat quality, reproduction, growth, production, disease resistance and immunity in goats and sheep from the literature. This may improve the results of genomic selective breeding. Genome-wide association study (GWAS) is a key technology to study the genetic basis of complex traits and diseases through genotype–phenotype association [35]. Ranran Liu et al. [2] developed a 55 K genotyping array and selected SNPs related to economic traits from the literature, which can be potentially applied to GWAS for traits of interest. Based on the genome sequencing data of cashmere goats, Xian Qiao et al. [15] added 858 SNPs of some genes that related to wool traits and designed a 66 K SHS-based target enrichment SNP chip for cashmere goats. It was successfully used for association analysis of cashmere fiber traits. Another method that can quickly find trait-associated SNPs is to search in the publicly available databases containing SNP and GWAS. It is known that GWAS Atlas is a manually collated resource of genome-wide variant trait associations for various species, involving cultivated plants and livestock (including goats) [36]. The continuous development and improvement of the AnimalQTLdb [37] also allows users to easily obtain QTL and SNP-gene association data on livestock species. Online databases can quickly find SNP sites associated with traits. However, we believe that the content of these databases is also based on published literature, which may be not comprehensive and requires regular updates over time. Although it is time-consuming and cumbersome to find SNP sites associated with traits by searching the literature, we can track newly discovered SNP sites associated with important traits.

Compared with the traditional single-locus genotyping method, cGPS is a kind of targeted sequencing genotyping technology. It uses capture probes to select DNA regions of great interest for high-depth sequencing analysis. Target-enriched SNP genotyping is a method with low cost and high efficiency. Targeted sequencing can not only obtain large-scale SNPs of different densities, but also provide more information on SNP variation, InDel and copy number variation [15]. This strategy of genotyping by targeted sequencing has many different names duo to the different methods of targeted enrichment and sequencing, such as SHS [38], GBTS [18], Target SNP-seq [39], MRASeq [40], etc. Among them, cGPS is a targeted sequencing genotyping technology of high and medium density (5 K-100 K target interval) that independently developed by Huazhi Biotechnology Co., Ltd., China.

To form the 10K cGPS liquid chip for Hainan black goat, we removed the repeated SNP sites and screened 10,677 qualified SNP sites from all 45,588 candidate sites. In general, the physical or genetic distance between markers and allele frequency are the main selection factors [16, 41]. According to the requirements of different chips, high-impact or rare variations, as well as variations of important traits, can be given priority. In this study, we also considered similar selection factors as described above. Finally, the sites on the 10K cGPS liquid chip were basically evenly distributed in autosomes. And only one in chromosome 6 had a high density distribution, which was a normal phenomenon. The sources of SNP sites on the 10K cGPS liquid chip are in line with our selection objective. SNP sites were mainly from resequencing data, followed by literature, and the least from GGVD. The annotation results of SNP sites on the chip showed that they were mainly located in the intergenic region and intron region. This was because that these SNP markers were designed to cover the entire genome. Meanwhile, it was also consistent with the annotation results of SNPs in our whole gene resequencing data. Most of the mSNPs (74.3%) in the 40 K maize mSNP panel developed by Zifeng Guo et al. were intergenic, 15.3% were in introns, and 6.2% were from other regions. We annotated the SNP sites on the chip and further associated the SNP sites with phenotypes. In the future, it can play an important role in gene mapping, GWAS, and molecular marker-assisted breeding of goat.

We verified the SNP sites detection rate, the consistency and repeatability of the genotyping results of the 10K cGPS liquid chip. The detection rate was between 97.34% and 99.93%. The repetition rate was between 99.66% and 99.82%. The consistency rate between cGPS liquid chip genotyping results and resequencing genotyping results ranged from 81.97% to 89.16%. The detection rate and repeatability of the chip were good, but the consistency rate of genotyping results was relatively low. We considered that different sequencing depths caused certain errors in the determination of genotyping results. Interestingly, one article has similar results with us. The verification results of the 200 K SNP array developed by Kang Wei et al. [42] showed that the average detection rate was 98.1%. The SNP repeatability of the repeated samples were 99.71% and 99.67%, respectively. The consistency rate of SNP genotyping between SNP array and resequencing data was 64.14%-91.93%, with an average of 84.07%. In order to further verify the accuracy of the array, they randomly selected inconsistent SNPs and performed sanger sequencing. The results showed that neither resequencing nor SNP array could guarantee 100% correct results [42]. Therefore, the subsequent mutual verification by different methods is very important.

If it is difficult to distinguish different breeds by phenotype, we should identify them at the molecular level. A southern Chinese goat breed with similar phenotype to Hainan black goat and a sheep breed were selected to verify the clustering ability of the chip. The results showed that 84.5% of the SNP sites were polymorphic and the heterozygosity rate was between 3.08% and 36.80%. It indicated that the 10K cGPS liquid chip can be used to determine the genetic variation of goat breeds in southern China. The chicken 55 K SNP genotyping array developed by Ranran Liu et al. showed 76.7%-88.0% SNPs were polymorphic in population verification [4]. The results of phylogenetic tree and PCA analysis showed that Hainan black goat, Yunshan black goat, Guizhou black goat and Small-tailed Han sheep were clustered to different positions, which basically realized the distinguishing function. The phylogenetic tree also showed that Hainan black goat in different regions of Hainan were not clustered but mixed together. The PCA results showed that the Hainan black goat populations were more dispersed, which was consistent with the phylogenetic tree results. This was because Hainan black goats in different regions of Hainan had not been breeded well by local farmers. In addition, the SNP genotyping data of the chip can also help to identify the pure Hainan black goat lineage, scientifically guide the hybridization and improvement of Hainan black goat, and contribute to the protection and development of goat germplasm resources. Hainan black goat cGPS chip is the first chip developed for tropical goat germplasm resources in China. Tropical goats closely related to Hainan black goat can also benefit from the chip.

Based on the results of the 10K cGPS chip for Hainan black goat, we counted the MAF of all SNP sites and analyzed the potential causes of a few low allele frequency variants. Variants with low allele frequency contain less information [16]. Among the total 10,677 SNP sites, a small number were found to have low allele frequency or even no polymorphism. For these SNP sites, we considered that there may be certain errors in probe design and the trait-related SNP sites found in the literature. Most of the samples we verified were Hainan black goats. Perhaps these variants did not exist in the samples we selected, but in other goat breeds. The design of 10K cGPS liquid chip is flexible. Therefore, if the SNP sites of the chip needs to be modified, more samples are needed to verify the feasibility of the adjustment. In this way, the liquid chip can be more suitable for the study of Hainan black goat and conducive to the conservation of Hainan black goat germplasm resources.

## Conclusions

In general, we realized the development and verification of the 10K cGPS liquid chip for Hainan black goat. For the design of the chip, goat resequencing data, GGVD and literatures were used to obtain candidate sites. A total of 10,677 representative SNP sites were selected to design probes, which covered 9,993 intervals and formed the 10K cGPS liquid chip. For the verification of the chip, the results showed that the detection rate of the sites was 97.34%-99.93%. Polymorphic SNP sites accounted for 84.5%. The heterozygosity rate was 3.08% -36.80%. The sequencing depth of more than 99.4% of sites were over 10X. Moreover, the repetition rate was 99.66%-99.82%. Due to the low depth of resequencing sites, the average consistency rate between chip genotyping results and the resequencing results was 85.58%, indicating that the genotyping results of the 10K cGPS liquid chip were more reliable. In addition, phylogenetic analysis proved that the chip had good clustering ability. The chip can accurately evaluate the genetic diversity of goats and provide a material basis for goat disease resistance breeding. Moreover, it can realize the breed identification and genetic relationship analysis of Hainan black goat, which lays a solid foundation for its subsequent breeding research.

## Materials and methods

**Fig. 7:**
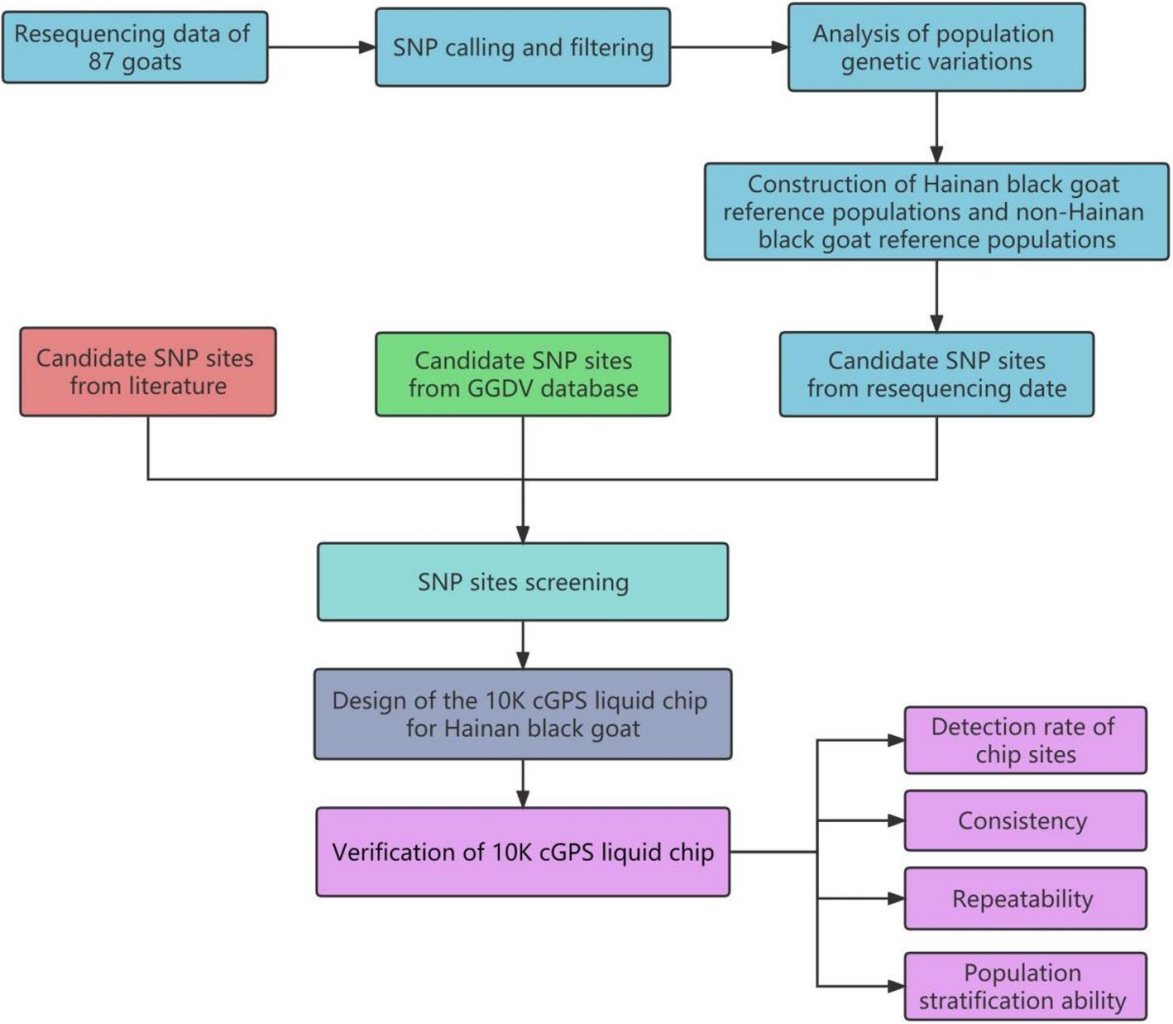
Road map for the development and validation of the 10k cGPS liquid chip for Hainan black goat

The source of SNP candidate sites is divided into three aspects. The development and verification roadmap of the 10K cGPS liquid chip for Hainan black goat is shown in Fig. 7. The establishment method of the 10K cGPS liquid chip is shown as below.

### Animals and DNA samples

A total of 104 DNA samples were used for the verification of the 10K liquid chip (Additional file 14: Table S14). The tissue samples or DNA samples of 104 samples were from 22 Yunshang black goats, 17 Guizhou black goats, 9 small-tailed Han sheep (DNA samples preserved by Chen Si, Hainan University), and 56 Hainan black goats from different regions of Hainan (including 16 re-sequencing DNA samples), respectively. In addition to the DNA samples previously preserved in our laboratory, the remaining DNA samples were extracted from freshly collected peripheral venous blood or ear tissues by using a genomic DNA extraction kit (Tiangen Biochemical Technology Co., Beijing, China) and stored at − 20 °C. The quality of DNA was detected by micro-spectrophotometer (IMPLEN GMBH Co., Germany) and 1% (w/v) agarose gel electrophoresis. The quality standards of DNA were as follows. Total DNA (without RNA) ≥ 1.0 μg (Qubit quantitative), concentration ≥ 20 ng/μL, volume > 50 μL, 1.8 ≤ OD 260/OD 280 ≤ 2.0, and OD 260/OD 230 ≥ 1.8. In electrophoretic detection, the main band of the sample was clear without degradation or slight degradation.

Blood samples and ear tissue samples of goats used in this study were collected under the supervision of veterinarians and were in accordance with the guidelines for experimental animals developed by the Ministry of Science and Technology (Beijing, China). It was also approved by the Animal Ethics Committee of the Institute of Animal Science (HNUAUCC-2022–00088). Neither anesthesia nor euthanasia was used. Clinical disease caused by sampling was not found in goats.

### Whole-genome resequencing data in goats and SNP calling

Whole genome resequencing (WGS) can detect a large number of SNP information through sequence alignment. Based on the detected variation information, liquid chip (cGPS) site can be developed. In order to obtain the whole genome resequencing data of goats, seven representative goat breeds were selected, including 6 Chinese local breeds (15 Longlin goats, 5 Leizhou goats, 16 Hainan black goats, 16 Dazu black goats, 15 Alxa cashmere goats, 10 Jining grey goats) and 1 foreign local breed (10 Boer goats). The data of 16 Hainan black goats were obtained from our previous sequencing results and uploaded to GenBank (accession number PRJNA754269) [43]. The whole genomes of the remaining 71 goat samples(15 Longlin goats, 5 Leizhou goats, 16 Dazu black goats, 15 Alxa cashmere goats, 10 Jining grey goats and 10 Boer goats) were from publicly available data downloaded from National Center for Biotechnology Information (NCBI, https://www.ncbi.nlm.nih.gov/).

To get high-quality SNPs for chip design, SNP calling was conducted as the following procedures. Sequencing data was filtered using fastp (v0.20.0) [44] and aligned to the goat reference genome (ARS1) by Burrows-Wheeler Aligner (v0.7.12-r1039) [45]. Picard (v1.107) was used to sort and convert sam files into bam files, and remove PCR duplicates [46]. GATK (v4.0.4.0) [47] was used to detect and filter SNPs. According to the genomic data, SNPs that meet the following criteria are retained. (1) Fisher test of strand bias (FS) ≤ 60. (2) Haplotype Score ≤ 13.0. (3) Mapping Quality (MQ) ≥ 40. (4) Quality Depth (QD) ≥ 2. (5) ReadPosRankSum ≥ -8.0. (6) MQRankSum > -12.5.

### Analysis of population genetic variations

In order to construct the reference population of Hainan black goat and non-Hainan black goat, population genetic and phylogenetic analysis were performed on 87 goat population samples and all samples, respectively. First, PLINK (v1.90) [48] was used to filter SNPs using the following criteria. (1) Remove the SNPs containing missing data points of > 10%. (2) Remove the SNPs with the minor allele frequency (MAF) value of < 0.05. After transforming the filtered-SNP sites into linear sequence information, the Neighbor-Joining (NJ) tree was constructed using MEGA-X [49] (Kimura 2-parameter mode and bootstrap for 1000 times). Finally, Rstudio (v4.0.5) was used to beautify the phylogenetic tree.

### Selection of candidate SNP sites from whole genome resequencing

Genetic differentiation index (Fst) is a method to measure the population differentiation and genetic distance, which is suitable for the comparison of diversity among subpopulations. The larger the differentiation index, the greater the difference [50]. VCFtools (v0.1.13) [51] was used to calculate the Fst value of each SNP variation site between Hainan black goat and non-Hainan black goat reference population. SNP sites with Fst > 0.5 were screened. The sites with high Fst value can be used to distinguish the genotypes of Hainan black goat and non-Hainan black goat populations.

The screening process of high polymorphic sites was described as below. VCFtools-0.1.13 was used to convert the original VCF file of resequencing SNP calling into plink (v1.90) format file. Afterwards, PLINK (v1.90) was used to screened the sites that met the following conditions. (1) MAF ≥ 0.05. (2) Missing rate ≤ 0.1. (3) Average depth (AV_ Deep) ≥ 2. Then, the hardy parameter in PLINK (v1.90) was used to calculate the heterozygosity of SNP sites. And the high polymorphic candidate SNP sites with observed heterozygosity (O HET) ≤ 0.3 were screened. High polymorphism sites can be applied to genotype analysis of different goat populations.

Goat genome variation database (GGVD, http://animal.nwsuaf.edu.cn/GoatVar) is dedicated to variation, selective characteristics and introgression regions [29] of modern and ancient goat genomes. It contains abundant information of goat genome variation. Goats include Bezoars, African goats, African dairy goats, European goats, Australian goats, Southwest Asian goats, South Asian goats, East Asian goats, Tibetan goats, Toggenburg goats, Saanen goats, Longlin goats, Leizhou goats, Cashmere goats, Beetal goats, and Bezoars vs. Domestic goats. The goat variation data, ARS1_SNPs.anno.tab.tar.gz (1.6G), was downloaded from GGVD. SNP sites with MAF value higher than 0.05 were screened, which reached up to 2,514 candidate SNPs. In addition, Hainan black goats have good disease resistance. Accordingly, SNP sites of immunogenes were specifically searched in GGVD, including 34 genes of interest such as *IL6*, *TNF*, *IL1B*, *IL10*, and *IFNG*. There are many SNP sites on all immunogenes. But GGVD does not have the variation data of Hainan black goat. Due to the close genetic relationship between Leizhou goat and Hainan black goat [44], we therefore focused on selecting the SNP sites in Leizhou goat when selecting candidate SNP sites of immunogenes.

### Selection of candidate SNP sites related to important traits from literature sources

Literatures was searched in PubMed (https://pubmed.ncbi.nlm.nih.gov/, accessed before 21 July 2021) and CNKI (https://www.cnki.net/, accessed before 21 July 2021). The search terms were goat SNP and sheep SNP in the both two websites. SNP that associated with goat or sheep traits were determined by browsing the title and abstract of the searched article. After carefully reading the literature, the SNP location information in the reference genome was recorded. Then the 101 bp base sequence containing 50 bp upstream and 50 bp downstream of the SNP site was searched on NCBI (https://www.ncbi.nlm.nih.gov/, accessed before 21 July 2021) or Ensembl (http://www.ensembl.org/, accessed before 21 July 2021). Afterwards, the sequence was aligned with the latest ARS1. And the SNP site was relocated to the position on ARS1. Therefore, we obtained a large number of trait-associated SNP site information, including the flanking sequence of SNP site, the genomic position of SNP site, reference and mutation of SNP, etc. Huazhi Biotechnology Co., Ltd verified whether the SNP sites and their flanking sequences could accurately correspond to ARS1, and finally determined the candidate SNP sites that related to important traits for chip development.

### SNP site screening principle and cGPS liquid chip design method

To meet the requirements of chip development, the collected SNP sites were screened. Firstly, the repeated SNP sites were removed. And the candidate SNP sites derived from resequencing data were preferentially selected as described below. (1) MAF > 0.1. (2) Missing rate < 0.1. (3) AV_Deep ≥ 2. (4) Heterozygosity rate ≤ 0.3. (5) SNP was the only variation type. Secondly, the high polymorphic candidate SNP sites from GGVD were filled and evenly distributed in the screened SNP sites derived from the resequencing data. Finally, the candidate SNP sites derived from literature and the SNP sites on the immunogenes of interest were added.

**Fig. 8:**
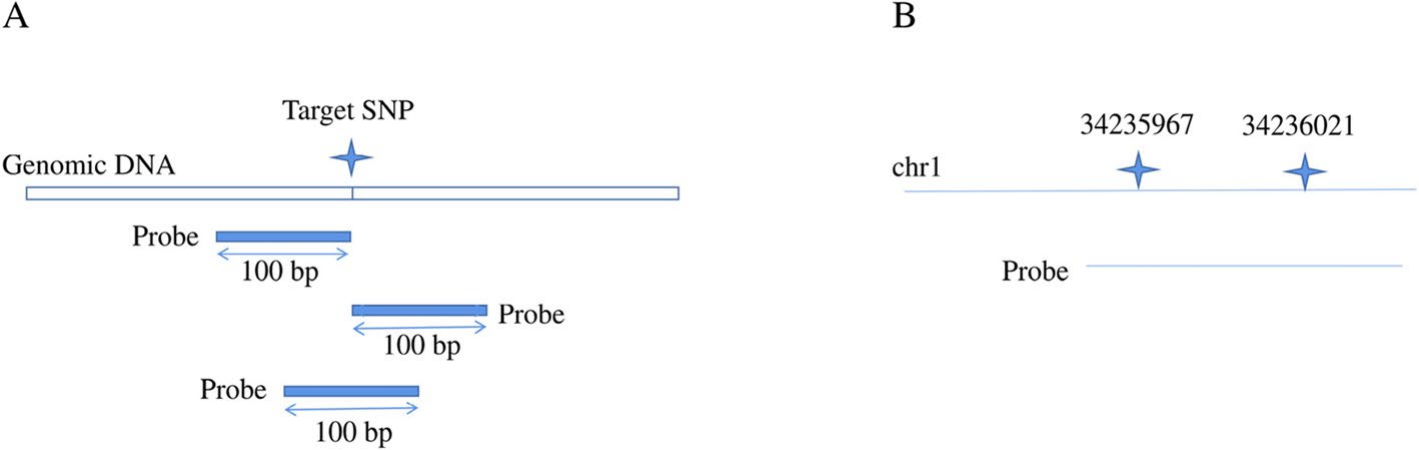
Schematic illustration of the probe design method. **A** Probe design for various cases of SNP site spacing. **B** Probe design for SNP locus spacing less than 100 bp

The probes were designed within the flanking sequence of the SNP site, which contained 100 bp upstream and 100 bp downstream of the SNP site (201 bp in total). Besides, the designed-probes should meet the following criteria. (1) The probe length was generally 100 bp (Fig. 8A). (2) The GC content of the probe was 20%-80%. (3) Single copy. (4) The number of SNPs in the probe coverage area was small. (5) The dimer and hairpin structure formed by the probe were in a reasonable range. (6) SNP sites were evenly distributed in chromosomes. Due to the short distance between some of the 10,677 SNP sites, the sites with a distance of no more than 100 bp can share a probe. For example, the two SNP sites, chr1:34235967 and chr1:34236021, had a distance of 54 bp and shared a probe as shown below (Fig. 8B).

The captured interval sequences are mainly analyzed by second-generation sequencing. To complete SNP calling and genotype analysis, it is necessary to align the obtained reads to a given region. The algorithm is as follows: take each SNP locus as the center and extend 100–200 bp upstream and downstream as the capture interval. For example, the corresponding capture interval of chr1:316747 was chr1:316597–316897. For two adjacent SNP sites, the overlapping region of the capture intervals can be taken as a new interval. For example, chr19:19216460 and chr19:19216471 shared a capture interval, which was chr19:19216320–19216631.

### Principle and application process of cGPS liquid chip

Genotyping by Pinpoint Sequencing of liquid captured targets (cGPS) is a high- and medium-density (5 K-100 K target interval) targeted sequencing genotyping technology independently developed by Huazhi Biotechnology Co., Ltd. Based on the optimized thermodynamic stability algorithm model, specific probes were designed for different target regions of the genome. The synthesized probes were used to capture and enrich multiple target sequences located in different genome locations by liquid-phase hybridization. After library construction and high-throughput sequencing, the genotypes of all SNP/InDel sites in the target region were obtained.

snpEFF 4.3t is an efficient software tool for functional annotation of detected gene variations. According to the location of the mutation site on the reference genome and the gene location information on the reference genome, the region of the mutation site in the genome and the influence of the mutation can be obtained.

After the development of the cGPS liquid chip, a large number of samples can be tested. The main steps were DNA extraction, library construction and quality control, liquid-phase hybridization and enrichment of target interval, second-generation sequencing, and bioinformatics analysis. Finally, variation analysis of the target interval in the tested sample was completed [18, 38].

### Verification of liquid chip

Firstly, in order to test whether each specific probe of the chip could locate to its target interval and accurately detect SNP sites, the SNP sites detection rate of the chip was verified. Through bioinformatics analysis of the chip sequencing data, the number of total SNP sites and polymorphic sites, detection rate, missing rate and heterozygosity rate of all samples on the cGPS liquid chip can be obtained.

In the second step, genotyping accuracy of the chip was evaluated, which contained consistency and repeatability verification. It is necessary to compare the genotyping results of SNP sites from the cGPS liquid chip with those from resequencing [25]. Therefore, we selected 15 samples of the Hainan black goat for cGPS liquid chip detection and acquired genotyping results of SNP sites from the chip. In addition, the previously obtained resequencing data was used to acquire genotyping results of SNP sites from resequencing. Then, the consistency of the two results (from cGPS liquid chip and resequencing, respectively) in each individual was evaluated. At the same time, four DNA samples were randomly selected, all of which was set up in duplicate. The results of two independently repeated detection in each sample was compared to verify the repeatability of the chip. If a locus was missing (NA) in one of the two results, then it would not be used for consistency or repeatability verification. A total of 108 detection results were obtained in this step, including 104 samples for consistency verification and 4 samples for repeatability verification. Among the detection data, MAF, Fst and other indicators of each locus were mainly selected for the above analysis.

Subsequently, the clustering ability of the chip was verified. We mainly focused on whether the chip can distinguish Hainan black goat from other breeds. DNA samples from 104 goats with definite breed were used for genotyping by the cGPS liquid chip. For the genotyping data of the samples, we deleted SNPs with call rate < 90% and MAF < 0.05 to ensure that the analyzed SNPs were in Hardy–Weinberg equilibrium (HWE) (*p* < 10^–6^) [52]. By using the filtered genotyping data, we applied MEGA-X for cluster analysis and iTOL (v4) for drawing the phylogenetic tree [53]. Besides, Python (v2) and smartpca were used to obtain the eigenvectors and eigenvalues. Finally, Rstudio (v4.0.5) was used to depict the principal component diagram.

### Statistical analysis

In order to process the data, EXCEL (v16.0.10338.20019) was used for calculation and drawing, and GraphPad Prism 8.0.2 was used to beautify the picture.

### Supplementary Information

**Additional file 1: Table S1.** Quality table of 87 goat resequencing data.

**Additional file 2: Table S2.** Information table of high polymorphism candidate SNP sites from resequencing.

**Additional file 3: Table S3.** Information table of candidate SNP sites with high Fst value from resequencing.

**Additional file 4: Table S4.** Information table of high polymorphic candidate SNP sites from GGVD.

**Additional file 5: Table S5.** Information table of candidate SNP sites of immune gene form GGVD.

**Additional file 6: Table S6.** Information table of candidate SNP sites related to important traits from literature.

**Additional file 7: Table S7.** Information table of SNP sites on the 10K cGPS liquid chip for Hainan black goat.

**Additional file 8: Table S8.** Microarray loci annotation results and GO and KEGG analysis of affected genes.

**Additional file 9: Table S9.** Statistical table of SNP detection rate in 104 samples.

**Additional file 10: Table S10.** Genotyping results and consistency rate of cGPS liquid chip and resequencing.

**Additional file 11: Table S11.** Information table of genotyping results in the repeated sample.

**Additional file 12: Table S12.** Information table of SNP sites genotyping data of the 108 samples detected by the chip.

**Additional file 13: Table S13.** Information table of SNP sites of the 108 samples detected by the chip.

**Additional file 14: Table S14.** Information table of 104 goat samples for chip verification.

**Additional file 15.** 108 goat chips test results.

## Data Availability

All data generated or analysed during this study are included in this published article and its supplementary information files.

## Abbreviations

cGPS: Genotyping by pinpoint sequencing of liquid captured targets
SNP: Single nucleotide polymorphism
WGS: Whole genome sequencing
GS: Genomic selection
GBS: Genotyping by sequencing
QTL: Quantitative trait sites
InDel: Insertion and deletion
ARS1: The goat reference genome
FS: Fisher test of strand bias
MQ: Mapping Quality
QD: Quality Depth
MAF: Minor allele frequency
NJ: Neighbor-Joining
Fst: Genetic differentiation index
AV_Deep: Average depth
O HET: Observed heterozygosity
GGVD: Goat genome variation database
NA: A locus was missing
HWE: Hardy-Weinberg equilibrium
GWAS: Genome-wide association study
